# Immune Landscape of Breast Cancers

**DOI:** 10.3390/biomedicines6010020

**Published:** 2018-02-11

**Authors:** Divya Nagarajan, Stephanie E. B. McArdle

**Affiliations:** Nottingham Trent University, Nottingham NG1 8NS, UK; divya.nagarajan@ntu.ac.uk

**Keywords:** breast cancer, TNBC, immune infiltrate, microenvironment, prognosis, microbiome, chemotherapy

## Abstract

Breast cancer is a very heterogeneous disease, both at a molecular and a histological level. Five intrinsic subtypes were initially identified—Luminal-A, Luminal-B, HER2^+^, Triple negative/basal like (TNBC) and normal like—subsequently expanded to seven (Basal-like-1 and 2, mesenchymal, mesenchymal stem-like, luminal androgen receptor, immuno-modulatory and unstable). Although genetic and epigenetic changes are key pathogenic events, the immune system plays a substantial role in promoting progression and metastasis. This review will discuss the extent to which immune cells can be detected within the tumor microenvironment, as well as their prognostic role and relationship with the microbiome, with an emphasis on TNBC.

## 1. Breast Cancer

Breast cancer is a very heterogeneous disease, both molecularly and histologically, with at least five intrinsic molecular subtypes [1,2]. These helps in determining the risk of progression, potential therapeutic resistance and even, for some, clinical outcome [3]. Knowing whether the tumor expresses a surface receptor or not has allowed for more precise therapeutic intervention thereby, significantly improving disease-free survival. Based on gene expression, profiling breast cancers are classified into major subtypes: ER^+^ mainly (+/-PR), HER2^+^ mainly, or devoid of any receptors, known as triple negative (TNBC). Each subtype has risk factors for incidence, treatment response, rate of disease progression and metastasis associated with it. Tumors expressing receptors for estrogen and progesterone generally respond well to hormonal interventions, while HER2^+^ tumors (which overexpress the ERBB2 oncogene) respond effectively when anti-HER2 therapy is used. Tumors that lack expression of all three receptors are very aggressive with no current molecular-based targeted therapies available. Moreover, only 20% of TNBC tumors respond well to neo-adjuvant chemotherapy, and those with residual disease after treatment have a significantly worse survival than those of different molecular subtypes [4]. Despite the many clinical trials using molecular/chemotherapeutic agents over the years, no drug compound has yet shown promising results for treating all TNBCs. Since then, TNBC has been further sub-classified into six distinct molecular features with unique sensitivity to therapeutic agents [5]. TNBC displays epidemiological and clinico-pathological features very distinct to the other subtypes. TNBC is associated with a higher incidence of relapses, which are early and visceral in location. In addition, despite a relative sensitivity to chemotherapy, prognosis remains poor.

## 2. Tumor Heterogeneity

**Figure 1:**
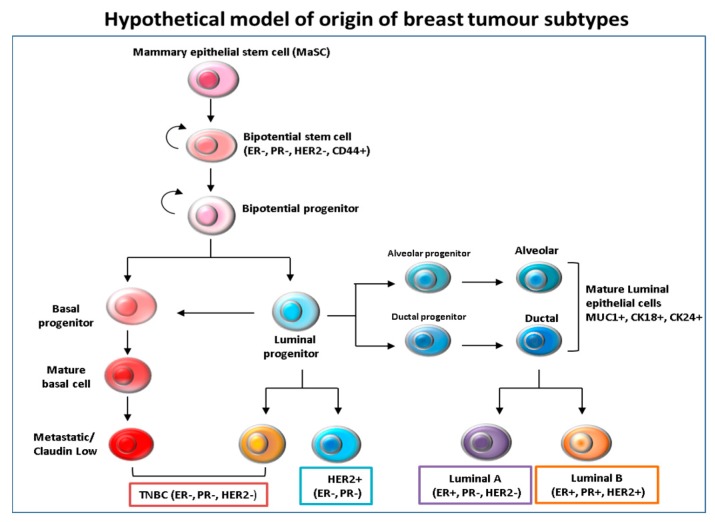
Hypothetical model of the origin and differentiation of breast cancer subtypes. It is believed that mammary epithelial stem cells (MaSc) represent the common cells of origin for all subsequent subtypes. The mammary stem cells then give rise to bi-potential stem cell progenitors, from which luminal and basal progenitor cells originate. The intermediate steps are driven by tumor subtype-specific transforming events that are still unclear. It is thought that basal progenitors can differentiate into basal claudin-low Triple negative/basal like (TNBC) subtypes, while luminal progenitors are likely to differentiate into both basal-like and luminal cells.

Tumor heterogeneity has been explained by several hypotheses, including subtype-specific tumor origin led by transforming events. Accordingly, ER^+^ and HER2^+^ have been proposed to emerge from luminal progenitors that are lineage-committed, whereas TNBC basal-like breast cancer subtypes have developed from under-differentiated stem cell-like progenitors. There is also evidence supporting the notion that luminal progenitors can switch phenotypes following epigenetic events, making them precursors for basal-like tumors [6] (Figure 1). To further complicate matters, heterogeneity has also been found within one tumor type where cells display various linvasiveness, angiogenic and metastatic traits [7]. As mentioned previously, besides the three major types of breast cancer, based on the gene expression profiles, TNBC have been further classified into six subtypes, namely, basal-like 1 (BL-1) and basal-like 2 (BL-2), an immune-modulatory (IM), a mesenchymal (M), a mesenchymal stem cell-like (MSL) and luminal androgen receptor (LAR) [5]. Another study, at Baylor University, classified TNBC into four distinct sub-types, with two subtypes based on whether the immune system is immunosuppressed (BLIS) or activated (BLIA), with the worst prognoses being associated with BLIS tumors [8].

## 3. Crosstalk between Immune Cells and Breast Cancer 

**Figure 2:**
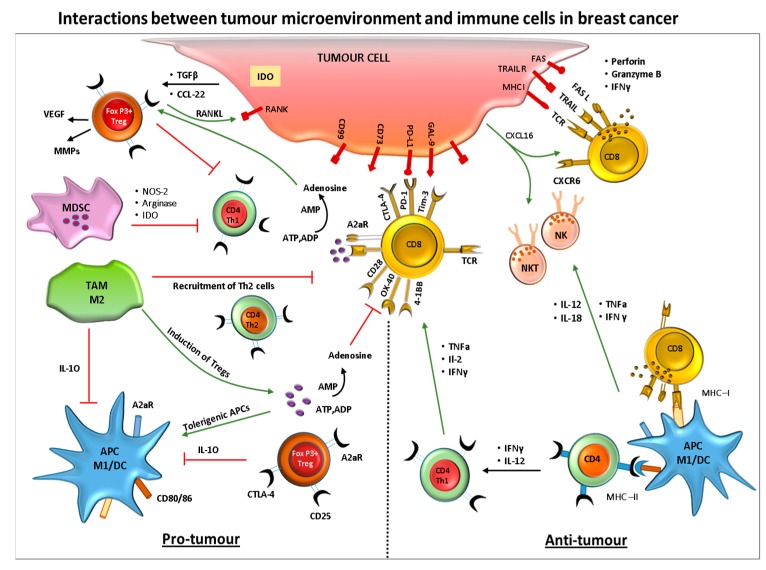
Crosstalk between breast cancer cells and cells of the immune system. The diagram represents the interactions between the tumor and immune cells during pro- and anti-tumor immune responses.

The exact mechanism by which breast cancer is initiated is unknown; however, it has been shown that during the development and progression of carcinogen-induced breast cancer, a significant immune-suppression, as detected by the inability of the cells to respond to proliferative agents, such as Con A and phytohaemagglutinin (PHA), has been linked to an increase in tumor incidence. This is due to dramatic atrophy of the thymus which results in a significant decrease in the overall number of T-cells, with reduced Interleukin -2 (IL-2) receptor expression [9]. More recently, it has been shown that the immune system plays a dual role in tumor initiation and progression, capable of both inhibiting or promoting tumor expansion. Cytokines, such as Transforming Growth Factor (TGF-β), Interferon γ (IFN-γ) and Tumour necrosis factor α (TNF-α), produced during the early stages of carcinogenesis taking place in an inflamed environment, will have anti-tumor action, while, when produced during chronic inflammation (i.e., once the tumor has been established), will actively promote growth and metastasis [10]. In breast cancer patients, prior to surgery and adjuvant therapy, a general immune system dysfunction favoring a Th2 response, was also found in comparison to healthy controls, as measured by the lower percentage of CD4^+^ and CD8^+^ T lymphocytes producing type 1 (lL-2, IFN-γ, or TNF-α) and type 2 (IL-4) cytokines [11]. Others have found that NK impairment also plays a role in the initial stages of human tumorigenesis in breast cancer [12]. The development of mice lacking not only T and B cells but also natural killer (NK) cells (NOD/SCID/gamma (null) (NOG) mice) further demonstrated the involvement of NK cells in tumor formation and spontaneous organ-metastasis [13]. In addition to this overall immune dysfunction, immune-suppressive cells, such as Tregs and myeloid-derived suppressor cells (MDSC), have been found in high numbers within the tumor microenvironment of breast cancers [14], playing a major role in preventing an effective endogenous immune response and the depletion of Tregs, even without any additional immunotherapy, is able to mediate a significant anti-tumor response. (Figure 2).

Moreover, some breast cancer tumor cells have been shown to be able to recruit MDSC via the mTOR pathway and the production of granulocyte colony-stimulating factor (G-CSF), which in turn promotes tumor progression and metastasis [15]. These suppressive cells stop T cell activation by pathways involving arginase, generation of reactive oxygen species (ROS) and NOS and T cell trafficking into tumor site. Upregulation of Indoleamine 2,3-dioxygenase (IDO) enzyme and arginase is also employed, to catabolize nutrients essential for the activation of effector T cells. Tumor cells also downregulate major histocompatibility complex (MHC) expression, antigenic expression and upregulate inhibitory signals, such as programmed death ligand-1 (PD-L1), to escape immune attack [16]. There is therefore no doubt that chronic inflammation influences the initiation, as well as the progression, of breast cancer, via the constant presence of inflammation related-cytokines and the recruitment of cells, such as Tregs and MDSC, which in turn diminish the little, if any, active immune responses.

## 4. Prognostic Values of Tumor Infiltrating Lymphocytes (TILS) in TNBC

Many immune cells can therefore be found either within the tumor itself, around the tumor or in close proximity to the tumor, namely in the stroma. Some will have anti-tumor activity while others will actively suppress the immune response. The exact nature of the cells present and their specific localization within this, so called, “immune contexture” has been shown to be extremely powerful with respect to their prognostic value in certain cancers [17]. Interestingly, although tumor infiltrating lymphocytes (TILs) were shown to correlate with pathological complete response (pCR) after neo-adjuvant chemotherapy (NAC) in all breast cancers, a significant correlation between TILs at diagnosis and overall survival was only observed in TNBC and HER2^+^ breast cancer; however, the reasons for this remain poorly understood [18,19].

In a recent literature search of twenty-five published studies comprising 22,964 patients, Mao Yan and colleagues were able to conclude that TILs had prognostic values only in TNBC for disease free survival (DSF) and Overall Survival (OS) [20]. A more favorable prognosis in patients with HER2^+^ breast cancer has also been reported [21], and in a large phase III randomized adjuvant breast cancer trial, assessing node-positive, ER^−^ /HER2^−^ BC, Loi S et al., found that high lymphocytic infiltration was associated with excellent prognosis [18]. Interestingly, although significant heterogeneity of this prognostic effect was observed in the subgroup made up of ER^+^ and ER^−^ subtypes, the presence of Treg was most consistently associated with poor prognosis, while the presence of CD8^+^ T cells and activated memory T cells was associated with a reduction in the risk of relapse in ER negative breast cancer [22].

## 5. Prognostic Values of TILS in HER2 Expressing Breast Cancers

The human epidermal growth factor receptor (EGFR) family comprises four trans-membrane receptors, which are involved in signal transduction pathways regulating cell growth and differentiation: EGFR/HER1, c-erbB2/HER2, HER3, and HER4 [23]. Breast cancers over-expressing the human epidermal growth factor receptors, HER1 (EGFR/c-erbB-1) or HER2 (neu-c-erbB-2), have been associated with disease progression, survival, stage and treatment response [24]. About one in five breast cancers express HER2 and half of these also express a steroid hormone receptor (either PR or ER), albeit less than HER2-negative, hormone-receptor-positive tumors and therefore, tend not to respond to tamoxifen treatment. These tumors also tend to be poorly differentiated, have high grades and have high proliferation rates. HER2 over-expression down-regulates HER1/EGFR associated downstream pathways [25]. Trastuzumab is a humanized monoclonal antibody (also known as Herceptin) against HER2, which, upon binding to HER2, is proposed to induce a series of events, such as inhibition of HER2 shedding, inhibition of the PI3K pathway, suppression of angiogenic factor vascular endothelial growth factor (VEGF) etc. However, these effects do not occur when normal levels of HER2 expression are present [26]. Overall survival and disease-free survival were both significantly improved in women with early and locally advanced HER2^+^ breast cancers treated with chemotherapy first followed by Trastuzumab, compared with women treated only with chemotherapy, while also increasing cardiac toxicity. Similar to TNBC, HER2^+^ breast cancers with over 50% of lymphocytic infiltration, both in and surrounding the tumors, have been shown to have better responses to chemotherapy and improved survival rates. Infiltration of >30% of breast tumors by CD8^+^, TBET^+^ T-cells (T-box transcription factor TBX21, a marker of type 1 T-cells) and more generally, Th1 type cells, can predict improved DFS [27], while the presence of Th2 and FoxP3^+^ are more generally associated with worse survival rates [28]. In TNBC, HER1/EGFR is frequently overexpressed (up to 80%), and anti-EGFR therapies, including small molecule tyrosine kinase inhibitors (TKI), have been used in the clinic with limited success so far. However, patients with high levels of CD8^+^ TIL have been shown to respond better to EGFR mAb neoadjuvant therapy [29]. Despite the correlation found between higher levels of EGFR and increased metastatic progression and decreased patient survival, metastatic TNBC appears to be unresponsive to EGFRi [30]. Interestingly, HER2 patients who have failed Trastuzumab are given a dual HER1/HER2 inhibitor, called lapatinib, to target multiple receptors of the HER family [31].

## 6. The Role of the Immune System in the Response to Chemo/Radiation Therapy

Breast cancers, especially basal-like, TNBC and HER2^+^, when infiltrated with immune cells have shown to be consistently associated with better prognoses, with or without any treatment. More specifically, stromal lymphocytic infiltration was shown to be a robust prognostic factor in TNBCs [19]. Pathological complete response (pCR) to neo-adjuvant chemotherapy remains the best predictor of disease progression for TNBC. Chemotherapy is currently the only treatment option for TNBC, and prolonged overall and event-free survival times have been shown to be associated with pathological complete response (pCR) to neo-adjuvant chemotherapy (NAC). In addition, regimens using sequential anthracyclines and taxanes in neo-adjuvant settings were shown to have a higher rates of pCR [32]. Basal-like breast cancers and TNBCs (which constitute more than 90% of all basal) are characterized as invasive ductal carcinomas of no special type, which are also known as invasive ductal carcinomas, not otherwise specified (IDC-NOS). Recently, Nakashoji et al., demonstrated that IDC-NOS with negative AR status and higher Ki-67 scores may be associated with chemotherapy sensitivity [33]. Immune infiltrates, found within the tumor prior to chemotherapy, are now regarded as being, at least partly, responsible for the response observed after chemotherapy because of their ability to induce an immune reaction against dying tumor cells as a consequence of the treatment.

**Figure 3:**
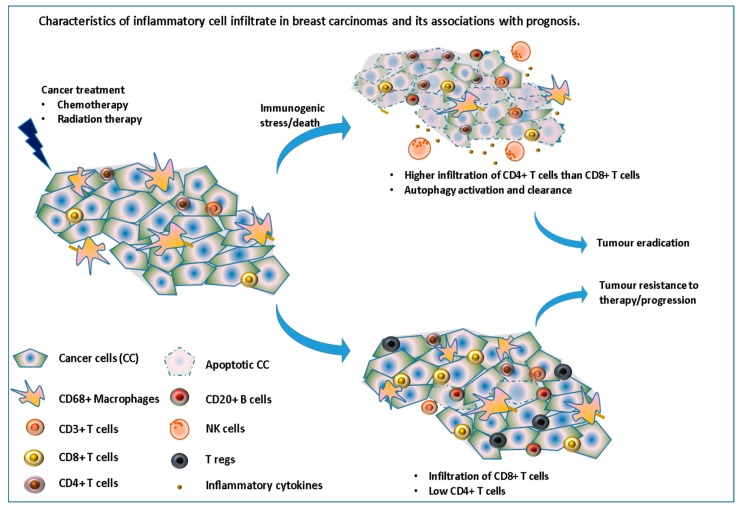
Immune cell infiltrate in breast carcinomas and its associations with prognosis. This illustrates the importance of inducing an immunogenic cell death via the recruitment and/or activation of many immune cells leading to tumor eradication.

However, this response depends on the type of cell death induced by the drug used. Kroemer’s group have previously shown how drugs, such as anthracyclines, can induce immunogenic cell death while others do not [34]. Moreover, in response to these therapeutic stresses, many cancer cells switch to a survival-promoting pathway called autophagy, where intracellular proteins and organelles in lysosomes are recycled, in order to provide substrates to sustain metabolism, while at the same time clearing the cells from these toxic accumulations of damaged proteins. Interestingly, autophagy has recently been shown to increase the immunogenicity of cancer cells by affecting their “adjuvanticity” rather than their antigenicity [35]. Chemotherapy can also decrease the number of regulatory T-cells and myeloid suppressor cells [36] as well as induce de-novo epitopes which, once taken and presented by the immune system, could stimulate a tumor-specific immune response. In a study assessing the immune infiltrate in 121 patients with stage II or III breast cancer, before and after receiving NAC, García-Martínez et al., reported that while a high number of CD3, CD4, CD68 and CD20 cells before treatment were significantly correlated with pCR after chemotherapy, mainly CD68^+^ cells and tumor associated macrophages were found to be associated with worse DFS and OS, especially for those with residual tumors (no pCR) [37] (Figure 3).

## 7. Future of Immunotherapy (IT) in Breast Cancer (BC)

As mentioned before, immune infiltrates are associated with better prognoses, mainly in HER2^+^ and TNBC breast cancer subtypes, and, while HER2^+^ cancer can be treated using an anti-HER2 antibody (Trastuzumab), no targeted therapy exists for TNBC. Moreover, with the promising efficacy of checkpoint inhibitors as monotherapies and in combination with chemotherapy for patients with cancers, such as melanomas, head and neck and lung cancers, researchers have studied TNBC to see whether such an approach could be used to treat TNBC. A study undertaken by Mittendorf et al., showed that 20% of TNBC express PD-L1 and that this expression was linked to PTEN loss [38]. Moreover, PD-L1 expression was found to be significantly more common in cancers with lymphocytic infiltrates [39], and PD-L1 expression on cancer cells was significantly correlated with PD-1 expression on TILs [40].

Both PD-1 inhibitor (pembrolizumab) and PD-L1 inhibitor (atezolizumab) have been assessed in early phase studies and due to their very promising results are now being assessed in many phase-III clinical trials to test their benefit in metastatic TNBCs, with or without chemotherapy [41]. In addition to PD1 and PD-L1, Indoleamine 2,3-dioxygenase-1 and -2 (IDO1,2) an intracellular enzyme overexpressed by many cancers, have also been shown to be involved in immune suppression via the depletion of tryptophan from the microenvironment and the production of metabolites, such as kynurenines [42]. Interestingly both IDO-1 and PD-L1 can be induced by pro-inflammatory cytokines, such as IFN-g, which further explain why initial, necessary and beneficial inflammatory responses are then followed by anti-inflammatory mechanisms when infection/tissue damage has been cleared/repaired, but are detrimental if prolonged due to chronic inflammation, such as in cancer. Moreover, the induction of IDO1 and therefore, kynunerine, can also lead to the induction of TGF-b and IL-6, which, in turn, further promotes the IDO1 expression thereby generating a positive feedback loop. It is therefore not surprising that tumors with high TIL infiltrate will also have an increase in the expression of PD-L1 and IDO. A recent study by Kim et al., where IDO1 protein expression was studied in 200 TNBC patients, found that over 50% of basal-like TNBC expressed IDO1 [43]. Epacadostat is an IDO1 inhibitor which has been shown to increase and restore the proliferation of dendritic cells, NK and T cells, but reduce Tregs. A clinical phase I/II study is currently ongoing, assessing the efficacy of combining Epacadostat and pembrolizumab (anti-PD-L1 antibody) in many cancers, including TNBC (Keynote 037-ECHO 202, NCT 02178722).

TNBC express antigens, such as NY-ESO1, MAGE-A, p53, and MUC1, which have the potential to be recognized by CD8^+^ T-cells, and many T-cell epitopes have been identified [44]. These can then be exploited to boost or generate a de novo immune response using dendritic cells, DNA or peptide with adjuvants as a delivery system or boost. Alternatively, more recent work has started using the patient’s own tumor as the source of antigens for a more personalized vaccine following neo-adjuvants where the genome of the patient’s own tumor is used to identify antigens relevant to the patient, and thereafter, some chosen peptides are synthesized in the form of long peptides with adjuvants such as Poly I:C (a TLR 3 agonist). A phase I clinical trial is currently ongoing to assess the feasibility of such a vaccine. Vaccines may also be the only hope for patients with residual TNBC disease post neo-adjuvant chemotherapy, since those have a worse prognosis than those presenting with non-TNBC. We have shown that TNBC patients with HAGE^+^ residual disease exhibited no TILs and had a two-fold death risk increase compared to HAGE- residual tumors [45].

## 8. Role of Microbiome in Breast Carcinogenesis

A person’s susceptibility to cancer is the result of the combined interaction between the person’s genes with the person’s own environment and lifestyle, which will directly influence the expression and modulations of many genes (epigenetics). These environment factors include Body Mass Index (BMI), physical activity, dietary habits, sleep, stress, environmental toxins, and hormonal imbalance [46]. Epigenetics refers to the alterations and modifications in gene expression and function without changing the actual DNA sequence. An individual’s epigenome can accumulate genetic alterations over a lifetime due to physical, environmental and/or undetermined factors. In breast cancer, epigenetic regulation changes in response to environmental stimuli that may often lead to inactivation or deletion of tumor-suppressor genes [47].

The microbiome is a frequent contributor to epigenetic dysregulation that interacts at both physiologic and environmental levels. The microbiome is a collective term for genes from microbial communities (fungi, viruses and bacteria) that are ubiquitous and reside in the oral cavity, upper and lower intestine, urinary tract, etc. The gut has a wide variety of bacteria that interacts with the immune system leading to downstream chemical changes involved in pathways that impact epigenetic alterations. A human body can host more than 10 trillion microbial cells that are believed to play important roles in an individual’s health. It is thought that humans have evolved a symbiotic relationship with large variety of bacteria residing in the gut and gastrointestinal tract, that contributes to the metabolism of food products [48]. The knowledge gained upon studying microbial communities is now being applied to understand the role of the microbiome in breast carcinogenesis and whether its manipulation can alter/influence breast cancer treatments.

Recently, the link between human gut microbial activity and levels of estrogen in the circulation and breast cancer risk have been studied [49] and the microbial composition was found to contribute to higher estrogen levels which, in turn, positively correlated with an increased risk of breast cancer.

The microbiome also contributes to tumorigenesis and metastasis by playing an active role in enhancing or undermining the host immune system. They contribute to chronic inflammation by modulating/maintaining gut epithelial cells and influencing the number of neutrophils, thereby enhancing or suppressing the immune system in both human and animal models [50]. As mentioned before, chronic inflammation is a well-established risk factor of tumorigenesis that produces toxins and free radicals that can damage host DNA and accelerate the development of mammary tumors [51,52].

Given the distant effect of the microbiome on organs, studies have focused on examining the colonizers of breast tissue. Besides the existence of microbiota in various parts of body, it has also been consistently found in breast milk, and Urbaniak and colleagues [53] postulated that bacteria use the nipple to gain access to breast ducts for creating a breast microbiome. Proteo-bacteria might be selected due their adaptability to high fatty acids in breast tissues, and other fat metabolizing bacteria, such as Enterobacteriaceae, Pseudomonas, and Steptococcus agalactiae, were also found to be present in breast tissues.

Xuan et al., 2014 compared the microbiome of ER^+^ tumors with normal tissues using next-generation sequencing analyses, and the results showed an increase in the presence of Methylobacterium radiotolerans, and a low number of Sphingomonas yanoikuyae [54]. It was postulated that the two bacterial strains balance the survival of each other in healthy tissues and a fall in the antibacterial response against tumor tissues was observed due to the decline in S. yanoikuyae, suggesting the probiotic function of the organism in the breast tissue [54]. These gram-negative bacteria express compounds, such as glycosphingolipid which activates natural killer cells and dendritic cells, and macrophages which are directly involved in the inhibition of tumor growth and the killing of tumor cells. The role of breast microbiota and the extent to which they help in shaping the immune response to provide a protective micro-environment or to promote tumorigenesis still remains to be studied.

Bacterial mechanisms can also impact epigenetic processes in response to environmental factors, by inducing changes to host signaling pathways. Epigenetic reprogramming that affects cancer cell-viability, migration, apoptosis, and gene expression can be induced by the over-production of bacterial metabolite by-products [55,56], through which they also gain resistance to antibiotics. This reprogramming has also been implicated in breast cancer subtype development, where the promoters of genes, such as ER α in TNBC [57] and BRCA1, have been found to be hyper-methylated [53].

Although bacterial influence can promote hyper-methylation and epigenetic reprogramming in humans, which leads to cancer, a direct cause of bacterial epigenetic activation inducing breast tumors formation remains to be proven. Epigenetic dysregulations which promote the development of breast cancers can also be fuelled by environmental factors and microbiome activities. The risk of cancer can be minimized by the establishment of a healthy microflora through specific dietary choices, which, in turn, will affect chronic inflammation, estrogen levels and obesity. It is well known that breast feeding by mothers helps to establish a diverse microbiome, thereby reducing the risk of obesity and helping to develop a healthy immune system [58,59]. Moreover, this early exposure to commensal microbes is also crucial for establishing mucosal tolerance, thereby protecting the individual from immune-mediated diseases, such as inflammatory bowel disease (IBD) and asthma during adulthood [60].

Understanding the role of microbiome can provide better insight into how to enhance the response to chemotherapeutic agents, e.g., cyclophosphamide. In addition, it has been shown that some products can be used to counteract chemotherapy side effects, such as diarrhea, thereby enabling a higher dose of drug to be given [61]. In addition, the use of probiotics can provide protection against pathogenic colonization and enhances mucosal physical barriers against invasion [58]. Moreover, certain bacteria, such as Lactobacillus acidophilus, have been shown to have the ability to reach the mammary gland [62] and possess anti-tumor properties [63]. Therefore, the knowledge gained from a person’s microbiome can be used to prevent or contribute to the fight against cancer and can even be manipulated for the development of breast cancer treatments.

## 9. Conclusions

**Figure 4:**
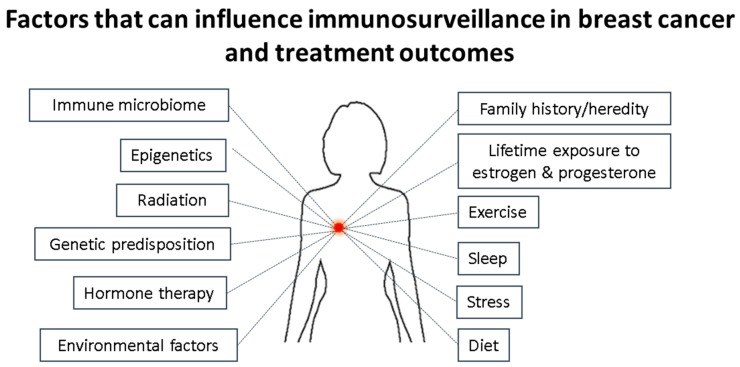
Factors influencing the immune surveillance in a breast cancer that can also impact the treatment outcome. This image shows that several non-biological and biological factors including host genetics and lifestyle factors can impact/affect the immuno-stimulatory and immunosuppressive tumor microenvironment thereby controlling the pathological complete response to a therapy/treatment.

Breast cancers are a group of heterogeneous diseases classified into several intrinsic molecular subgroups based around the expression of either estrogen, progesterone, over-expression of HER2, alone or in combination, or none of these. The expression or over-expression of these have allowed targeted therapies to take place, thereby drastically improving the outcomes of many patients. However, patients suffering from the breast cancer type not expressing any of these or those who have failed to respond to the targeted approaches can only be treated with chemotherapy, and while this works well at first, many cancers relapse or continue to progress. More recently it has been found that the immune system plays a critical role in the initiation, progression of the tumor and/or response to treatments. The manipulation/targeting of inhibitory immune molecules has already revolutionized the outcome of many cancers and is now being investigated for the treatment of breast cancer in particular TNBCs. Cancer is the results of years of cycles of damaged healing processes at cellular levels, led by chronic inflammation and influenced by the genetic make-up (predisposition) of the person. The difficulty will therefore be to induce strong anti-tumor immune responses using a combination of immune stimulation and checkpoint inhibitors, while, at the same time, attempting to switch from chronic tumor promoting inflammation to a more acute anti-tumor type of inflammation. However, doing so without taking into account the microbiome (influenced by the diet and exercise of the person) and mental state of the person (stress, sleep, undealt emotional load) may very well be counter-productive, knowing the influence all these have on the immune system (Figure 4).
